# Downregulating miRNA-199a-5p exacerbates fluorouracil-induced cardiotoxicity by activating the ATF6 signaling pathway

**DOI:** 10.18632/aging.205679

**Published:** 2024-03-25

**Authors:** Wei Wang, Liang Dong, Hengxu Lv, Yonghui An, Changwang Zhang, Zheng Zheng, Ying Guo, Li He, Libin Wang, Jinmei Wang, Xinlei Shi, Na Li, Mingqi Zheng

**Affiliations:** 1 Department of Oncology, The First Hospital of Hebei Medical University, Yuhua, Shijiazhuang 050031 Hebei, China; 2 Department of Cardiology, The First Hospital of Hebei Medical University, Yuhua, Shijiazhuang 050031, Hebei, China; 3 Hebei Key Laboratory of Heart and Metabolism, Shijiazhuang 050031, Hebei, China

**Keywords:** miRNA-199a-5p, ATF6 signaling pathway, fluorouracil, cardiotoxicity

## Abstract

Background: Fluorouracil (5-FU) might produce serious cardiac toxic reactions. miRNA-199a-5p is a miRNA primarily expressed in myocardial cells and has a protective effect on vascular endothelium. Under hypoxia stress, the expression level of miRNA-199a-5p was significantly downregulated and is closely related to cardiovascular events such as coronary heart disease, heart failure, and hypertension. We explored whether 5-FU activates the endoplasmic reticulum stress ATF6 pathway by regulating the expression of miRNA-199a-5p in cardiac toxicity.

Methods: This project established a model of primary cardiomyocytes derived from neonatal rats and treated them with 5-FU *in vitro*. The expression of miRNA-199a-5p and its regulation were explored *in vitro* and *in vivo*.

Results: 5-FU decreases the expression of miRNA-199a-5p in cardiomyocytes, activates the endoplasmic reticulum stress ATF6 pathway, and increases the expression of GRP78 and ATF6, affecting the function of cardiomyocytes, and induces cardiac toxicity. The rescue assay further confirmed that miRNA-199a-5p supplementation can reduce the cardiotoxicity caused by 5-FU, and its protective effect on cardiomyocytes depends on the downregulation of the endoplasmic reticulum ATF6 signaling pathway.

Conclusions: 5-FU can down-regulate expression of miRNA-199a-5p, then activate the endoplasmic reticulum stress ATF6 pathway, increase the expression of GRP78 and ATF6, affect the function of cardiomyocytes, and induce cardiac toxicity.

## INTRODUCTION

Fluorouracil (5-FU fluorouracil, 5-FU) is an anti-metabolic cycle drug for tumors, which mainly inhibits S-phase cells. 5-FU has a definite curative effect and strong anti-tumor effect and is the main drug for colorectal cancer, head and neck cancer, gastric cancer, pancreatic cancer, and breast cancer [1, 2]. However, the increase in survival rate has made people realize the importance of improving 5-FU-induced cardiotoxicity during treatment [3, 4]. In real clinical practice, to improve the therapeutic effect, an increase in 5-FU dosage might cause an increase in cardiac toxicity. 5-FU-caused cardiotoxicity is often progressive and irreversible, which severely limits its widespread and long-term clinical use [5, 6]. The incidence of 5-FU cardiotoxicity reported in the literature ranges from 1.2% to 18.0%, and the related mortality rate ranges from 0% to 8%. The differences in incidence rates may be related to the type, usage, dosage, sample size of 5-FU drugs [4]. Therefore, to some extent, the effectiveness of 5-FU is limited. Analyzing and clarifying the mechanisms that lead to cardiac toxicity is of great significance for drug application and management in the clinic.

Rezkalla et al. [7] reported that 68% of patients who use 5-FU experience characteristic ischemic changes in their electrocardiogram. 5-FU can directly lead to myocardial cell damage and ischemia [8]. The involved pathogenesis needs further clarification. The damage to myocardial cells may lead to the accumulation of toxic metabolites [4], which directly affects the cell cycle, mitochondrial energy metabolism, and endoplasmic reticulum (ER) stress [9]. ER stress plays a key role in the damage of myocardial cells. The sensors of ERs include activating transcription factor 6 (ATF6), inositol-requiring enzyme type 1 (IRE1), and protein kinase R (PKR)-like endoplasmic reticulum kinase (PERK) [10]. ATF6 acts as a nodal regulator of proteostasis in ER [11] by inducing the major molecular chaperones, such as glucose-regulated protein 78 (GRP78), glucose-regulated protein 94 (GRP94) and CHOP [12, 13]. One study shows that JAK2 or STAT3 inhibitor reduces 5-FU resistance and autophagy through ATF6-mediated ER stress in gastric cancer cells, which also enhances the sensitivity of gastric cancer cells for 5-FU [14].

The microRNAs (miRNA) participate in regulation of oxidative stress and myocardial cell injury [15–17], and they participate in regulating apoptosis. Dysregulation of miR-199a-5p contributes to multiple cardiovascular diseases [18]. Chen et al. [19] reported hsa-miR-199a-5p protect hypoxia induced injury of myocardial cells. Other studies [20, 21] also shown the protective role of miRNA-199a-5p under hypoxic conditions. The current study aims to explore the role of a miRNA-199a-5p splicing form, miRNA-199a-5p, in 5-FU induced cardiotoxicity, further exploring its underlying mechanism, especially whether the interaction between miRNA-199a-5p and ATF6 contributes to its cardio beneficial effect.

## MATERIALS AND METHODS

### Animal model and grouping

The study was conducted on healthy male Sprague Dawley (SD) rats (weighing 220-250 grams), and total two batches of experiments were conducted. For the first batch, 18 rats were randomly divided into a control group, a low-dose (25 mg/kg) 5-FU group, and a high-dose (50 mg/kg) group, with 6 rats in each group. The control group was given physiological saline, and the two model groups were established by given different doses of 5-FU solution, which was injected intraperitoneally once every other day and 7 times in a row. This batch experiment was conducted to investigate the cardiac injuries caused by 5-FU, and expression of miRNA-199a-5p and ATF6.

The second batch was used to study the therapeutic effect of miRNA-199a-5p on cardiac injury caused by 5-FU *in vivo.* The miRNA-199a-5p mimic and its control sequence was prepared into 0.5% mass concentration and finally 0.1% oligonucleotide liposomes mixed with 40 mg/L cationic liposomes were obtained. The mixed liposomes were injected into the caudal vein of rats with concentration of 4 μg/g. body weight 2 days after 5-FU administration (50 mg/kg) and twice a week with 5 times in a row.

### Cardiac function assessment

The cardiac toxicity induced by 5-FU is mainly characterized by a decrease in myocardial contractility [22, 23]. The left ventricular structure and function are good indicators for evaluating cardiac damage. Therefore, the subsequent structural and functional analysis was performed in the left ventricle of rats. The cardiac function was measured using The Vevo 770 (Visualistics Inc., Toronto, Canada) ultrasound application and was operated by ultrasound medical professionals.

### Histopathological analysis by Hematoxylin and Eosin staining and immunohistochemistry

The rats were anesthetized with 3% pentobarbital (0.2 ml/100 g. body weight) and were fixed on the small animal operating table. After abdominal disinfection, the abdominal wall was cut to expose the abdominal cavity. The rat’s chest was quickly opened, and the heart was removed for protein content measurement and staining. The myocardial tissue was immersed in 4% paraformaldehyde for 24 hours to fix it. After gradient dehydration, embedded it in paraffin and prepared 6 μm tissue slices. The slices were stained by Hematoxylin and Eosin (HE) staining. To quantitatively evaluate the damage of 5-FU to myocardial tissue, the injury score of myocardial tissue was analyzed following the methods reported by Oei et al. [24] and Ghafoor et al. [25]. The criteria for score include contraction bands/coagulation necrosis (0 for absent; 1 for 1%–10% of cardiomyocytes; 2 for 11%–50% of cardiomyocytes; 3 for 51%–100% of cardiomyocytes); interstitial edema (0 for absent and 1 for present); granulation infiltration (0 for absent, 1 for present <50%, and 2 for present≥50%); platelet aggregates/thrombi (0 for absent and 1 for present) and extravasation of red blood cells (0 for absent and 1 for present).

Immunohistochemistry was used to detect the ATF6 expression in cardio tissues. After dewaxing and antigen repair, the sodium citrate antigen repair solution (P0081, Beyotime, China) was added on slices. The slices were cultured in 100° C water bath for 10 minutes, washed by PBS solution, and then cultured in PBS solution containing 0.5% Triton X 100 and 1% BSA. The primary antibody for ATF6 (PA114886, 1:100 diluted, Invitrogen, USA) was added and cultured overnight at 4° C, after being blocked by 10% goat serum for 1 hour. Subsequently, goat anti mouse IgG (1:50, Beyotime, China) was added dropwise and incubated at 37° C for 40 minutes. The 3.3ʹ-diaminobenzidine (DAB) substrate (DAKO, Glostrup, Denmark) were used to visualize antigen presence.

### Isolation and culture of primary cardiomyocytes from newborn rats

The 5-FU treated cell model was established using primary myocardial cell isolated lines from neonatal rats. According to method reported by Maas et al. [26], newborn SD rats (Weitong Lihua Experimental Animal Technology Co., Ltd., Beijing, China) were euthanized with small scissors. The rats were disinfected with 75% alcohol, and the left ventricle was dissected by squeezing the heart. The heart was quickly placed in precooled PBS solution and cut into pieces. Tissues were digested with a mixture of 0.05% trypsin and 0.05% collagenase for 5 minutes. After precipitation, the supernatant was discarded. After repeating the above operation 3 times, the mixture was centrifuged at 1500 rpm for 5 minutes, and the supernatant was removed. The isolated cells were cultured in DMEM-F12 medium (11320033, Gibco) containing 15% FBS 2 hours. The differential adhesion method [27] was used to separate and isolate types of cells. The cells were cultivated in DMEM/F12 medium (11320033, Gibco, Paisley, UK) for 24 hours until spontaneously contracted cells can be seen under an optical microscope (Nikon Company, Tokyo, Japan).

### Measurement of cell viability

Cells were cultured in an incubator containing 5% CO_2_. The logarithmic growth phase of cells suspension was inoculated into a 6-well plate (2 mL/well). Before the experiment, cells were cultured with serum-free DMEM-F12 for 24 hours to synchronize the cell cycle. The control group only added the 5-FU solvent dimethyl sulfoxide (DMSO), while the drug group added 5-FU (200 μmol/l, 400 μmol/l, F8300, Solarbio Life Science, China) and cultivated for 48 hours to harvest cells. The logarithmic growth primary cardiomyocytes were inoculated into 96 well plates with a concentration of 1×10^4^ cells/well. Ten microliters of CCK8 solution (C0042, Beyotime, China) was added to cells and cultured for another 4 hours. The absorbance value of each well at 450 nm was measured by a microplate reader and the cell viability rate (%) was calculated according to the absorbance value of the treatment group and the control group.

### Quantitative reverse transcription PCR (qRT-PCR)

The total RNA was extracted from tissues or cells using TRIzol reagent (15596026, Invitrogen, USA). According to the instructions of the TaKaRa kit (RR037A, TaKaRa, Japan), the extracted RNA was reverse transcribed into cDNA in 10 μl reactions following the protocols provided by the manufacturer. A real-time fluorescent qPCR reaction system for PCR was prepared using the SYBR Green method (RR420, TaKaRa, 20 μl reaction mixture) in Applied Biosystems® 7500 system (Foster, CA, USA). The relative expression of miRNA-199a-5p was determined by 2 ^- ΔΔ Ct) method, according to normalization of U6 snRNA expression data. GAPDH was adopted as an internal reference for ATF6.

Primers for miRNA-199a-5p: 5’-CCGGGATCCGCAAACTCAGCTTTAC-3’ and 5’-CGGAATTCGTGGCGACCGTGATACC-3’; U6: 5’-CTCGCTTCGGCAGCACA-3’ and 5’-AACGCTTCACGAATTTGCGT-3’; ATF6: 5’-TATCCCTCCACCTCCATGTCA-3’ and 5’- TCTCGATTTGGTCCTTTCCACT-3’; GAPDH: 5’-GTATCGGACGCCTGGTTAC-3’ and 5’-CTTGCCGTGGGTAGAGTCAT -3’.

### Measurement of MDA, SOD, GSH/GSSG ratio, and LDH level

The primary cardiomyocytes that received different treatments were collected. Cell lysate (P0013, Beyotime, China) was added to lyse cells on ice for 30 minutes according to the manufacturer’s instruction. The lysed cells were centrifuged at 4° C for 12000 r/minutes for 15 minutes, and the supernatant was collected and measured according to the protocols provided by the manufacturer. MDA, SOD, GSH/GSSG, and lactate dehydrogenase (LDH) were measured by the commercial kits (S0131S, S0101S, S0053, C0017, Beyotime, China) by ELISA methods respectively. For rats in a different group, the abdominal aorta blood was drawn and centrifuged. The SOD activity, GSH/GSSG ratio and LDH level in serum were measured.

### Vectors

To explore the roles of ATF6 on cardiomyocyte, the cDNA sequence of ATF6 (https://www.ncbi.nlm.nih.gov/gene/22926) were amplified and extracted. The adenoviral vector containing full-length of ATF6 (Ad-ATF6) was inserted to the Xba I site of pAAV-CMV vector (6230, TaKaRa Bio, Japan) of AAVpro® Helper Free System (6651, TaKaRa). The recombinant vector was co-transfected to AAVpro 293T cells with pRC6 vector, and pHelper vectors. The cells were collected and lysed 3 days after transfection. Gradient ultracentrifugation was adopted to purify AAV6 viral particles. After AAV6 particles were quantified by real-time PCR, the particles were introduced to cardiomyocyte cells. The sequence of hsa-miRNA-199a-5p was obtained from miRBase (https://mirbase.org/) and the sequence is: CUUGUCCAUCAGACUUGUGACCC. Its mimic (miR10000232-1-5) and its negative sequence (miR1N0000002-1-5) were obtained from Guangzhou RiboBio Co., Ltd., and transfection was performed according to standard reported methods.

### Western blot

The previous protocols were used as previous publications {Shi, 2023 #13868}. The myocardial tissue was cut into pieces and lysed by RIPA lysate. Homogenize thoroughly with a homogenizer and centrifuge 12000 g for 15 minutes at 4° C. After determining the protein concentration using the BCA method, 50 μg protein samples were loaded and separated by sodium dodecyl sulfate-polyacrylamide gel electrophoresis (SDS-PAGE), after PVDF conversion, 5% skimmed milk powder was added at room temperature and cultured for 2 hours, then the solution was removed and the primary antibody for ATF6 (PA114886, 1:100 diluted, Invitrogen), GRP78 (ab21685, 1:1000 dilution, Abcam), CHOP (PA5-102305, 1:1000 dilution, Invitrogen), PERK (PA5-15305, 1:1000 dilution, Invitrogen), were added, and the second antibody (G-21234, Invitrogen) was added, the membrane was cultured overnight in a 4° C refrigerator. The bands were developed through ECL reagent (P0018M, Beyotime) and analyzed via the Image Lab Software (Version 6.0, Bio-Rad Laboratories, Hercules, CA, USA).

### Statistics

The SPSS 20.0 statistical software (IBM, Chicago, IL, USA) was used for data analysis. All data are presented by GraphPad 9.3.1 software. All *in vitro* studies were repeated in triplicate. The student’s t-tests were used for the two-group comparison with normal distribution data. the ANOVA analysis was adopted for multiple groups comparison followed Newman-Keuls post-hoc comparisons. The Spearman’s correlation analyses were used for correlation analysis. P<0.05 was statistically significant.

### Data availability

The data are available from the corresponding author on reasonable request.

## RESULTS

### The 5-FU results in cardiomyocyte damage and impaired cardiac function

**Figure 1:**
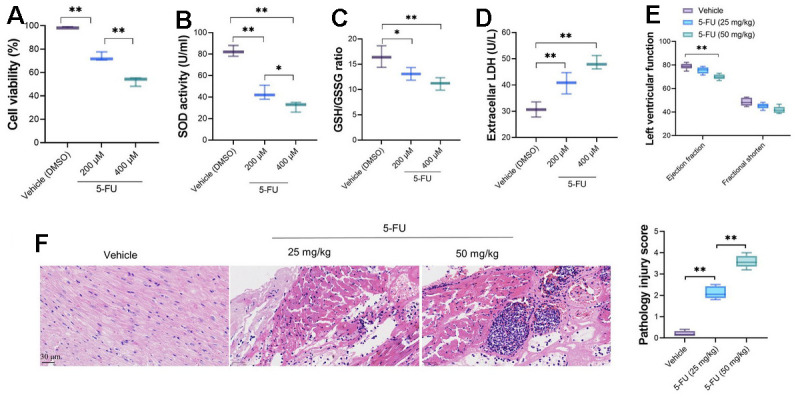
**5-FU causes cardiomyocyte damage and impaired cardiac function of SD rats.** Two doses, 200 and 400 μM were used for *in vitro* analysis and 25 and 50 mg/kg were adopted for *in vivo* analysis. (**A**) 5-FU decreases the viability of cardiomyocytes in both doses; (**B**–**D**) 5-FU causes decreasing of SOD activity and GSH/GSSG ratio, and increases LDH levels in primary cardiomyocytes; (**E**) 5-FU did not cause obvious impaired left ventricular cardiac function in rats, although it led to a downward trend; (**F**) HE staining indicates that 5-FU caused myocardial injury, which demonstrated as intermuscular inflammatory cell infiltration and focal necrosis in myocardial tissue.

The effect of serial concentration of 5-FU on primary cardiomyocytes was measured by CCK-8 assay. 5-FU decreased the variability of primary cardiomyocytes significantly (Figure 1A); moreover, the viability of cardiomyocytes decreases with the increasing of 5-FU concentrations (200 μM and 400 μM). The measurement of SOD activity revealed a significantly decreasing in 5-FU treated cardiomyocytes (Figure 1B), as well as decreased GSH/GSSG ratio (Figure 1C) and increased LDH levels (Figure 1D). Although the cardiac function measured by transthoracic echocardiography showed that compared vehicle group, 5-FU did not cause obvious impaired left ventricular cardiac function in rats (Figure 1E). HE stains revealed that 5-FU caused myocardial injury, which demonstrated contraction bands and granulation tissue with collagen deposition and neovascularization in myocardial tissue with higher pathology injury score (Figure 1F).

### miRNA-199a-5p is down-regulated in 5-FU treated cardiomyocytes and attenuates toxicity caused by 5-FU

**Figure 2:**
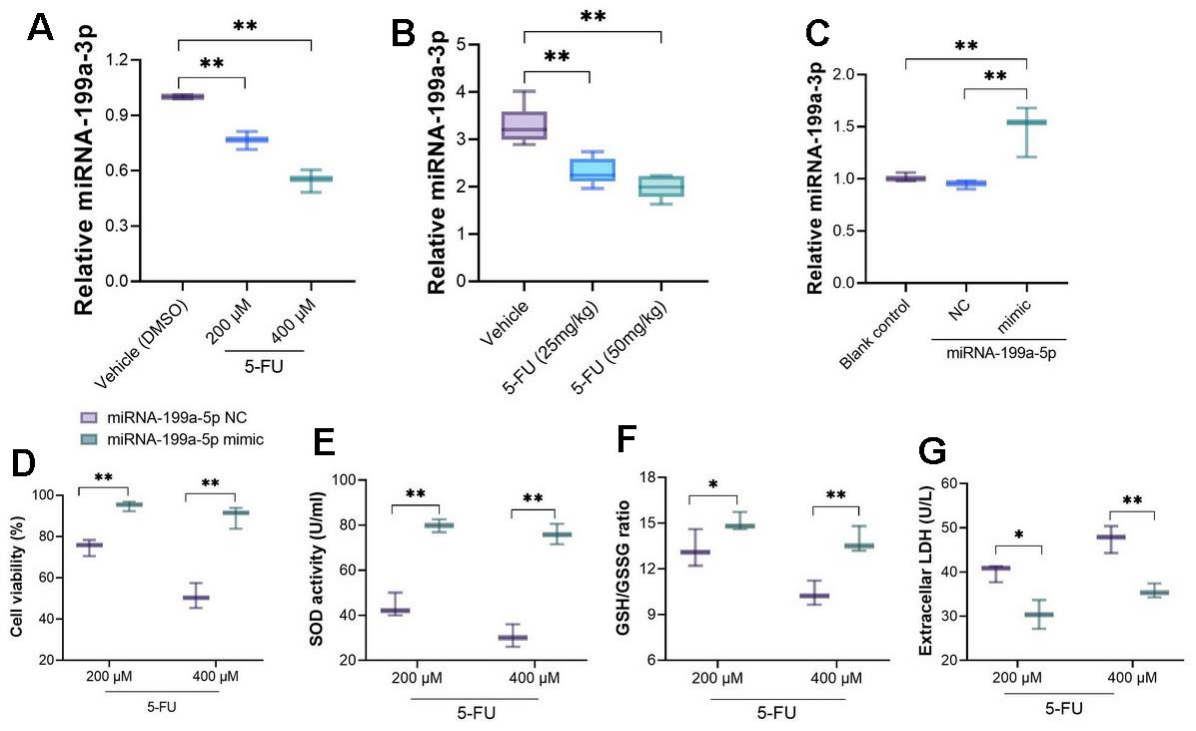
**5-FU treatment leads to down-regulated miRNA-199a-5p in cardiomyocytes and myocardial tissue.** (**A**) miRNA-199a-5p was down-regulated after primary cardiomyocytes were treated with 5-FU; (**B**) down-regulated miRNA-199a-5p could be observed in 5-FU-treated rats when compared with rats receiving vehicle treatment; (**C**) the miRNA-199a-5p mimic was introduced to primary cardiomyocytes, RT-PCR was performed to measure the transfection efficacy; (**D**–**G**) the upregulation of miRNA-199a-5p improved the cell viability caused by 5-FU; increased GSH/GSSG ratio and SOD activity, decreased LDH levels compared with miRNA-199a-5p NC.

RT-PCR analysis showed that miRNA-199a-5p was down-regulated after primary cardiomyocytes were treated with 5-FU (Figure 2A). *In vivo* analysis also demonstrated down-regulated miRNA-199a-5p in heart tissues of 5-FU-treated rats when compared with rats receiving vehicle treatment (Figure 2B). To clarify whether miRNA-199a-5p mediates the changed function of cardiomyocyte cells, we transfected miRNA-199a-5p mimic to cells and confirmed the successful transfection (Figure 2C). The upregulation of miRNA-199a-5p improved the decreasing cell viability (at 48h) caused by 5-FU. Also, the upregulation of miRNA-199a-5p increased GSH/GSSG ratio and SOD activity, decreased LDH levels in 5-FU treated cells when compared with miRNA-199a-5p NC (Figure 2D–2G).

### ATF6 is activated in 5-FU treated myocardial tissues and a potential target for miR-199-5p

**Figure 3:**
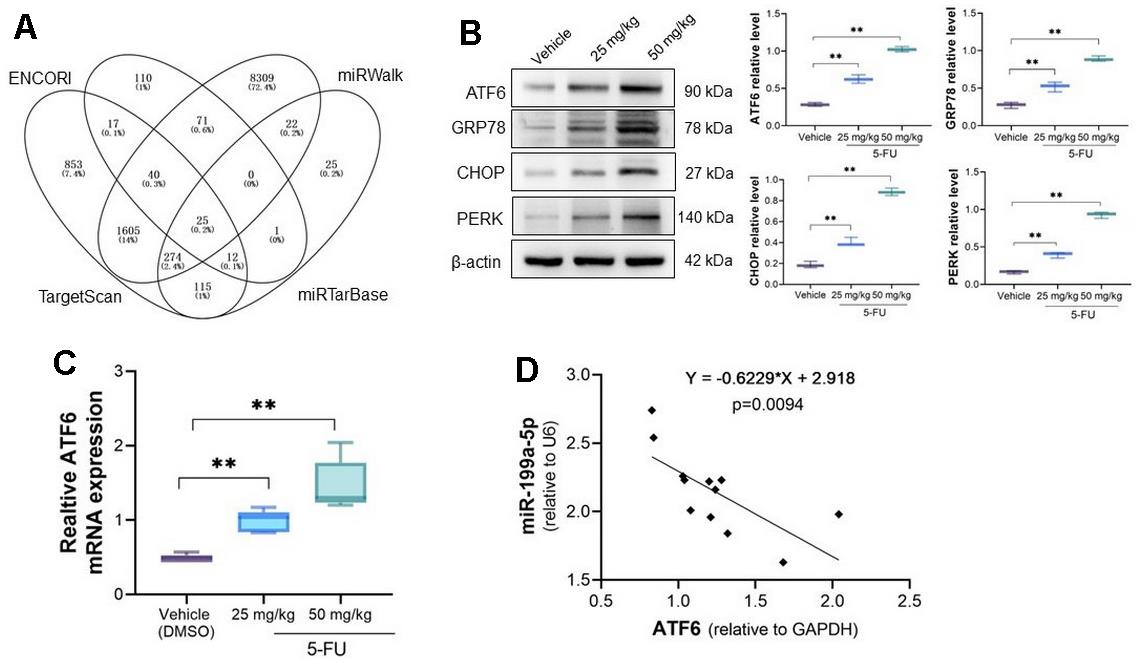
**ATF6 is activated in 5-FU treated myocardial tissues.** (**A**) The potential target of miRNA-199a-5p were explored by online tools and ATF6 was confirmed has potential binding sites with miRNA-199a-5p; (**B**) the expression of ATF6, GRP78, CHOP, PERK protein increased in 5-FU treated cardiomyocytes tissues; (**C**) the qRT-PCR analysis of ATF6 expression in cardiomyocytes; (**D**) the correlation of ATF6 and miRNA-199a-5p by RT-PCR analysis. ATF6 is negatively correlated with miRNA-199a-5p in 5-Fu treated myocardial tissues.

Bioinformatics analysis of potential targeted gene were analyzed by online tools ENCORI (https://rnasysu.com/encori/index.php), miRWalk (http://mirwalk.umm.uni-heidelberg.de/), TargetScan (https://www.targetscan.org/vert_72/), and miRTarbase (https://mirtarbase.cuhk.edu.cn/~miRTarBase/miRTarBase_2019/php/index.php). A total of 25 common potential binding target genes were obtained. ATF6 (Activating transcription factor 6), an endoplasmic reticulum protein that alleviate myocardial injury, was noticed and has possible binding sites with miRNA-199a-5p (Figure 3A). Yao et al. indicated that 5-FU causes ER stress in breast cancer cells [28]. Our analysis indicates ATF6 expression increased in 5-FU treated myocardial cells and tissues (Figure 3B, 3C). We also observed increased GRP78, CHOP, and PERK as well as ATF6 in myocardial tissue (Figure 3B). The correlation analysis demonstrates that ATF6 is negatively correlated with miRNA-199a-5p in 5-Fu treated myocardial tissues (Figure 3D).

### miRNA-199a-5p attenuates 5-FU induced toxicity in cardiomyocytes by targeting ATF 6

**Figure 4:**
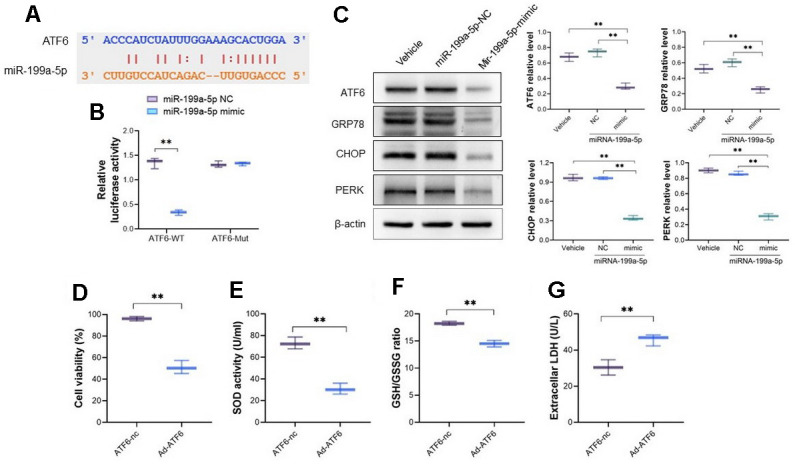
**miRNA-199a-5p attenuates 5-FU induced toxicity in cardiomyocytes by targeting ATF 6.** (**A**) The bioinformatics analysis indicates the binding sites between ATF6 and miRNA-199a-5p; (**B**) the dual luciferase reporter assay was used to verify the binding between miRNA-199a-5p and ATF6. The miRNA-199a-5p mimic decreased the luciferase activity of cells transfected with vector containing wild-type ATF6 sequence; (**C**) the cardiomyocytes cells were pretreated with miRNA-199a-5p, and the protein expression of ATF6, GRP78, CHOP and PERK decreased in 5-FU stimulated cells; (**D**–**G**) the adenoviral vector encoding full-length of ATF6 (Ad-ATF6) was introduced to cardio myocyte cells. The increasing expression of ATF6 decreased cell viability, SOD activity, GSH/GSSG ratio and alternatively increased LDH level in cardiomyocyte.

The potential targeted sites of miRNA-199a-5p and ATF6 were listed in Figure 4A and the dual luciferase reporter assay was used to verify their binding by constructed plasmid containing mutant ATF6 sequence. The miRNA-199a-5p mimic decreased the luciferase activity of cells transfected with vector containing wild-type ATF6 sequence, but not mutant ATF6 sequence (Figure 4B), which confirmed their direct binding. After cardiomyocytes cells were pretreated with miRNA-199a-5p mimics, the protein expression of ATF6, GRP78, CHOP and PERK decreased in 5-FU stimulated cells (Figure 4C). On the other hand, the adenoviral vector encoding full-length of ATF6 (Ad-ATF6) was introduced to cardiomyocyte cells. The cell viability, SOD activity, and LDH were measured. It follows that increasing expression of ATF6 decreased cell viability, SOD activity and GSH/GSSG level, and alternatively increased LDH level in cardiomyocyte (Figure 4D–4G).

### miRNA-199a-5p attenuates 5-FU induced toxicity via ATF 6

**Figure 5:**
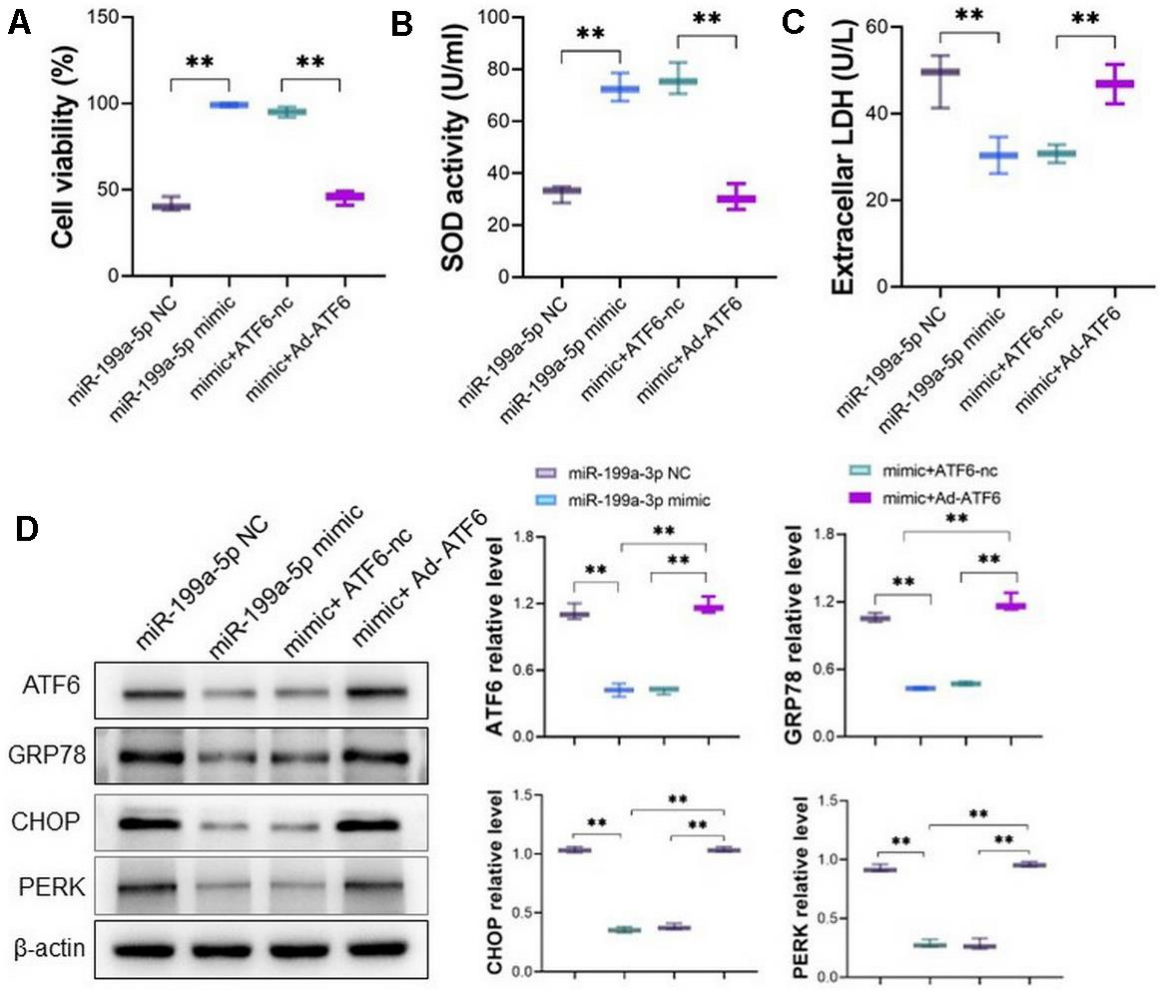
**miRNA-199a-5p attenuates 5-FU induced toxicity via ATF6 *in vitro*.** (**A**–**C**) The mimic of miRNA-199a-5p and Ad-ATF6 were co-transfected into cardiomyocytes, and the miRNA-199a-5p mimic could not attenuate 5-FU induced toxicity by cell viability, SOD activity, GSH/GSSG ratio and LDH level; (**D**) Western blot analysis indicates ATF6 was downregulated along with downregulated expression of GRP78, CHOP and PERK in cardiomyocyte.

To verify the effect of miRNA-199a-5p decreasing 5-FU induced toxicity is relying on ATF6, the mimic of miRNA-199a-5p and Ad-ATF6 were co-transfected into cardiomyocyte. Consistent with our predictions, overexpression of ATF6 abolished the protective effect of miRNA-199a-5p mimic attenuating 5-FU (400 μM) induced toxicity (Figure 5A–5C). Western blot also confirmed overexpression of ATF6 reversed the decreasing expression of ATF6, GRP78, CHOP and PERK in cardiomyocyte caused by miRNA-199a-5p (Figure 5D).

### miR-199a-5p attenuates cardiac injuries in rats caused by 5-FU through down-regulation of ATF6 *in vivo*

**Figure 6:**
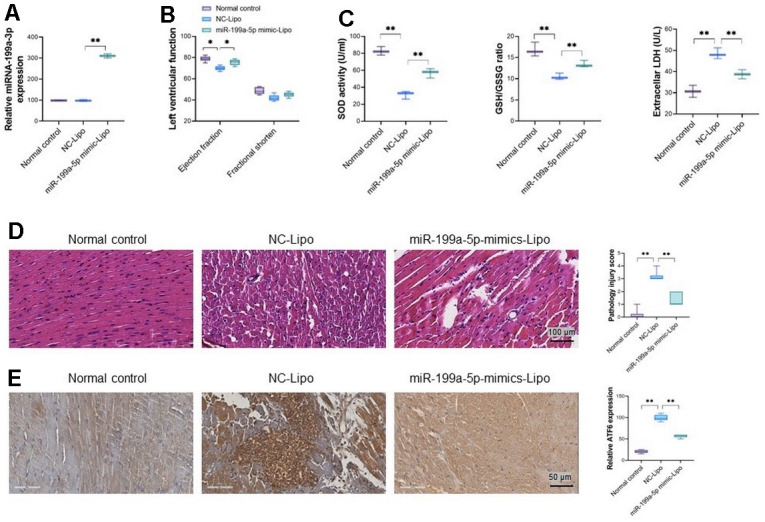
**miRNA-199a-5p attenuates 5-FU induced toxicity via ATF6 *in vivo*.** (**A**) miRNA-199a-5p in heart tissue after exogenous introduced; (**B**) miRNA-199a-5p liposomes improves left ventricular developed pressure in 5-FU injured heart tissue. (**C**) miRNA-199a-5p liposome decreases LDH, increases SOD activity and the ratio of GSH/GSSG in injured cardiac tissues; (**D**) the pathological injuries in cardiac tissues caused by 5-FU ameliorated by miRNA-199a-5p liposome; (**E**) miRNA-199a-5p liposome treatment reduced ATF6 expression in injured cardiac tissues significantly. The expression of ATF6 was confirmed by immunohistochemistry analysis.

To verify the therapy potential of miRNA-199a-5p against cardiomyocyte injury caused by 5-FU *in vivo* (50 mg/kg), cationic liposomes carrying miRNA-199a-5p and NC sequence were given to the 5-FU treated rats via caudal vein. The significantly increased level of exogenous miRNA-199a-5p in the heart tissue was confirmed in gene-delivery rats by RT-PCR and shown in Figure 6A. Compared with NC-Lipo group, obviously improved heart function could be observed in miR-199a-5p group, which demonstrated as increased left ventricular ejection fraction (Figure 6B). Meanwhile, the miRNA-199a-5p liposome decreased LDH, increased SOD, and the ratio of GSH/GSSG in heart tissues of 5-FU treated rats, compared with Lipo-NC group (Figure 6C). The miRNA-199a-5p liposome treatment also ameliorated the pathological injuries in cardiac tissues caused by 5-FU (H &E staining, Figure 6D), which was manifested by reducing the rupture of partial myocardial cells or decreased the necrosis of myocardial contraction bands. Immunohistochemistry demonstrated that miR-199a-5p liposome treatment significantly reduced ATF6 expression in injured cardiac tissues (Figure 6E).

## DISCUSSION

Currently, the exact pathogenesis of cardiac toxicity caused by 5-FU is not yet clear. The coronary artery spasm and secondary myocardial ischemia caused by 5-FU are considered as the main pathogenic mechanisms [8]. In animal models, 5-FU can induce coronary artery vasoconstriction mediated by protein kinase C pathway [29]. As a cytotoxic drug, 5-FU can directly damage vascular endothelial cells and caused secondary myocardial ischemia; on the other hand, 5-FU increases the peroxidation reaction (nitric oxide) synthesis of cell membrane lipids, as well as the intracellular oxidative stress response mediated by molecular reactive oxygen species [30], leading to endothelial cell damage and apoptosis. 5-FU metabolite alpha fluor beta alanine and its downstream product fluoroacetate are known cardiac toxic substances [31]. Moreover, 5-FU decreases the levels of myocardial mitochondrial ATP and mitochondrial membrane potential, and aggravated myocardial fibrosis and cardiomyocyte apoptosis [32].

miRNA is a type of noncoding small molecule RNA composed of 22 nucleotides. They regulate cellular biological behavior through activation of downstream signaling pathways. miRNA-199a-5p is a newly discovered miRNA [33] and reported dysregulated in multiple cardiovascular diseases [18, 19]. In a cardiac hypertrophy rats’ model, Yan et al. [34] exogenously upregulated miRNA-199a-5p levels and reported restored mitochondrial structure and function in rats. They indicated that miRNA-199a-5p regulates mitochondrial fatty acid oxidation and oxidative phosphorylation via the PGC-1α/ERRα axis and their downstream pathway. The above evidence suggests that miRNA-199a-5p is an important regulatory factor for oxidative stress in cardiac cells.

ERs are involved in the occurrence of cardiovascular diseases, including atherosclerosis, ischemic cardiomyopathy, hypertension, and heart failure [35]. Blocking the activation of the apoptotic pathway in ERs and the key links in resisting stress damage are important directions for research and treatment of various cardiovascular injuries. Many injury disease models have found that ATF6 and/or ERs signal pathways are elevated or activated. These tissue injuries are often accompanied by apoptosis, indicating that ERs related apoptosis pathways are activated [36, 37]. ATF6 is a member of the activating transcription factor/cyclic adenosine monophosphate effector element binding protein (ATF/CREB) family. In response to a cellular stressor resulting in the accumulation of misfolded proteins, the activation of ATF6 under ERs is dependent on BiP (also known as GRP78) [38]. Upon activation of UPR, ATF6 is released and binds to the promoters of UPR target genes, such as BIP/GRP78, GRP94, CRYAB, and XBP1, and VEGF [39, 40]. ATF6 also acts as the regulatory factors of early apoptosis activating CHOP in the nucleus [41]. This study reveals that 5-FU induces down expression of miRNA-199a-5p and increases ATF6, GRP78, CHOP and PERK expression. This indicates that ERs are involved in 5-FU induced cardiotoxicity, and ERs and its regulatory genes/products can be potential therapeutic targets. Bioinformatics analysis shows that downregulation of miR199a-5p leads to upregulation and activation of ATF6. The exogenously increased miRNA-199a-5p attenuates the injury introduced by 5-FU. Moreover, increasing ATF6 abolishes effect of miRNA-199a-5p.

**Figure 7:**
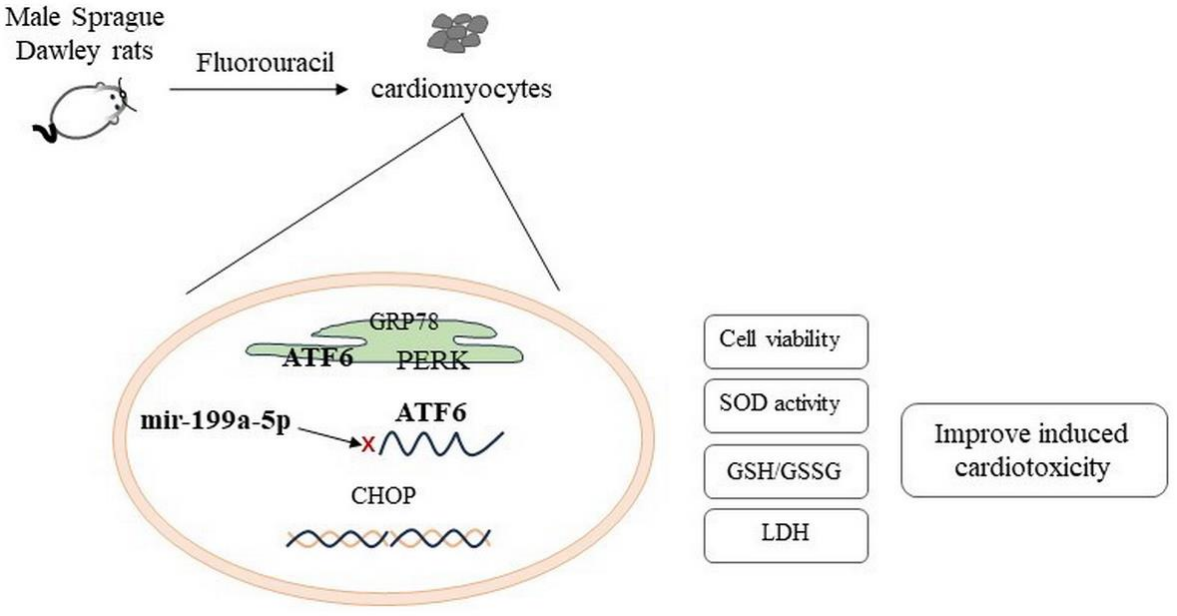
5-FU causes the decreased expression of miRNA-199a-5p and activated the ATF6 and PERK in endoplasmic reticulum stress, which led to the up regulation of CHOP expression.

On the other hand, activated UPR response by ATF6 to ERs also has the function of protecting cells. In a mouse heart fibrosis model, upregulation of ATF6 can inhibit fibrosis and enhance cardiac function. In cardiac myocytes, ATF6 induces GRP78 to restore ER proteostasis [42]. Knock out ATF6 causes cardiac fibroblast activation [43]. Our study showed that 5-FU causes the decreased expression of miRNA-199a-5p activated the ERs ATF6 pathway, which led to the up regulation of GRP78, CHOP, PERK and ATF6 expression affecting the function of cardiomyocytes and inducing cardiac toxicity (Figure 7). It is further confirmed that the cardiac toxicity caused by 5-FU can be alleviated after miRNA-199a-5p is up-regulated, and the protective effect depends on the down-regulation of endoplasmic reticulum ATF6 signaling pathway.

Our research conclusion is preliminary, from a therapeutic perspective, the introduction of exogenous miRNAs into the rats through tail vein injection. This treatment method should be carefully considered in the future, including whether other organs could be affected by miRNAs and how effective the treatment is in the actual human body. To sum up, 5-FU will produce certain cardiac toxic reaction in the process of anti-tumor treatment. Under the condition of toxic stress, the expression level of miRNA-199a-5p is significantly reduced, activating the endoplasmic reticulum stress ATF6 pathway, affecting myocardial cell metabolism and antioxidant activity, leading to cardiomyocyte damage, and inducing cardiac toxicity.
